# High frequency of pre-existing neutralizing antibody responses in patients with dengue during an outbreak in Central Brazil

**DOI:** 10.1186/s12879-016-1867-6

**Published:** 2016-10-07

**Authors:** Angela Ferreira Lopes de Teive e Argolo, Valéria Christina de Rezende Féres, Marli Tenório Cordeiro, Lucimeire Antonelli da Silveira, Adriana Oliveira Guilarde, Ernesto Torres de Azevedo Marques, Wayner Vieira de Souza, Celina Maria Turchi Martelli

**Affiliations:** 1 Institute of Tropical Pathology and Public Health of Federal University of Goiás, Rua 235, St Universitário, Goiânia, 74.605-050 GO Brazil; 2 Public Health Laboratory of Secretaria Estadual de Saúde de Goiás (LACEN-GO), Av. Contorno, 3.556, Jd. Bela Vista, 74.853-120 Goiânia, GO Brazil; 3 Pharmacy College of Federal University of Goiás, Av. Universitaria, St. Universitario, 74.605-220 Goiânia, GO Brazil; 4 Centro de Pesquisas Aggeu Magalhães da Fundação Oswaldo, Av. Prof. Moraes Rego, 1235, Cidade Universitária, 50.670-901 Recife, PE Brazil; 5 Center for Vaccine Research, Department of Infectious Diseases and Microbiology, University of Pittsburgh, Pittsburgh, PA USA

**Keywords:** Dengue virus, Neutralizing antibody, Dengue fever, Brazil

## Abstract

**Background:**

This study aims to identify dengue neutralizing antibody response in patients with dengue from a well-characterized cohort during an outbreak in central Brazil, 2012–2013.

**Methods:**

We analyzed paired samples from 40 patients with severe dengue and 20 patients with dengue. Eligibility criteria were: IgM, NS1Ag and/or RT-PCR positivity and positive IgG result. Plaque reduction neutralization test (PRNT_50_) from DENV-1 to DENV-4 was performed to identify serotype-specific NAbs response. An infecting serotype was defined as ≥4-fold increase in DENV NAbs in paired samples. Monotypic response was classified as PRNT_50_ ≥ 1/20 to only one DENV serotype, and multitypic response was considered to be PRNT_50_ ≥ 1/20 to two or more serotypes simultaneously.

**Results:**

Patients were mainly adults. Virological dengue infection was confirmed by RT-PCR: DENV-4(*n* = 14) and DENV-1(*n* = 10). Forty-four out of 60(73.3 %) patients had NAbs to DENV-4, DENV-1(68.3 %), DENV-2(68.3 %) and DENV-3(61.6 %) respectively. Fifteen percent of the cases presented monotypic response, whereas 85 % had multitypic response. DENV-4 infected-patients presented the greatest difference in PRNT_50_ titers compared with other serotypes. Pre-existing DENV NAbs was not correlated with disease severity. This was the first time that DENV-4 was implicated in an epidemic in the region.

**Conclusion:**

Our data indicates high exposure of multiple DENV serotypes in all age groups in the pre-dengue vaccine era and also previous to Zika virus introduction in Brazil.

**Electronic supplementary material:**

The online version of this article (doi:10.1186/s12879-016-1867-6) contains supplementary material, which is available to authorized users.

## Background

Dengue is one of the most relevant arboviruses in the world, endemic in more than 120 countries in tropical and subtropical areas [1]. Dengue virus is a mosquito-borne flavivirus with four characterized antigenically distinct serotypes (DENV-1, DENV-2, DENV-3 and DENV-4). Approximately 390 million dengue infections occur every year with ~100 million symptomatic illnesses according to a recent modeling estimation [2]. In the American region, nearly 61 % of the 2.38 million dengue cases were reported in Brazil in 2013 [3]. Nationwide there was evidence of an upward trend in the number of reported dengue cases, hospitalization and deaths in the last decade [4–6]. In central Brazil the first dengue outbreak was registered in 1994 with the detection of DENV-1, followed by DENV-2 (1998), DENV-3 (2002) and DENV-4 (2011) [7, 8]. Over the last two decades the peak of dengue transmission occurred in 2013 with ~55 thousand cases registered with an incidence rate of 4411 per 100,000 inhabitants [9].

Dengue may develop as an unapparent infection with the majority of cases presenting mild disease, but life-threatening conditions, death may occur [1]. Susceptible individuals mount a specific immune response when they are infected by one of the four DENV serotypes. Sequential infections also occur after exposure to different serotypes [10, 11]. DENV infection is believed to induce a long-lasting protective immunity to homologous serotypes. In addition, due to cross-protection, a transient immunity to heterologous serotypes may play a beneficial role in clinical outcomes [12]. By contrast, the viral infection mediated by antibodies (ADE) may be amplified during a secondary infection with a heterologous serotype, leading to deleterious immune response and disease severity [13–15].

The plaque reduction neutralization test (PRNT) is currently the gold standard to identify dengue serotype-specific neutralizing antibodies. This assay allows the detection of pre-existing and/or current DENV infection and seems to be correlated with protection [16]. In the current study, we aimed to identify the DENV neutralizing antibody response in paired samples collected from patients with dengue enrolled in hospital and ambulatory settings in central Brazil. The objective was also to assess the correlation of pre-existing DENV neutralizing antibodies by PRNT with dengue outcomes during the 2012–2013 outbreak. The study was conducted alongside the first DENV-4 outbreak, examining simultaneous co-circulation of the four serotypes in the region. The antibody profile is crucial to understanding the risk factors for disease severity and the protective role of the forthcoming dengue vaccines.

## Methods

### Study population

A clinical cohort of 452 laboratory-confirmed dengue cases was assembled in the city of Goiania in central Brazil (1.3 million inhabitants) [17] during the dengue outbreak of 2012–2013. The study area also has endemic yellow fever with high vaccination coverage (17DD yellow fever) in the population [18]. We recruited cases in eight public health clinics and in one public and two private hospitals. Briefly, patients were clinically and epidemiologically evaluated at baseline alongside with collection of blood samples at acute phase of disease (≤7 days of onset of symptoms) (t0). The follow-up consisted of a clinical evaluation and blood collection during a recent convalescence at 8–15 days (t1) and late convalescence at 20–30 (t2) days after the onset of symptoms. The patients were clinically classified as dengue, dengue with warning signs (DWS) or severe dengue (SD) according to the World Health Organization criteria [1].

For the current study, dengue cases were defined by at least one of the following tests: IgM, NS1Ag and/or RT-PCR. At least one positive IgG result in paired sera was required to perform PRNT.

Out of the entire cohort, we selected the first 40 patients who were diagnosed with either DWS (dengue with warning signs) or SD (severe dengue), considering the previously defined eligibility criteria. As a comparison group, we also selected the first 20 enrolled dengue eligible patients.

### Laboratory procedures

Blood samples collected during the clinical evaluation (t0, t1 and t2) were processed, and sera were cryopreserved according to biosafety guidelines at the Laboratory of Molecular Biology and Immunology of Infectious Diseases of Federal University of Goias in Goiania, Brazil.

We performed the following tests at baseline and follow-up: hematocrit, platelet, aspartate aminotransferase (16-50U/L), alanine aminotransferase (19–41 U/L) and albumin (3.5–5.0 g/dL); normal ranges reported in parentheses. For hematocrit 10 % increase in paired samples was defined hemoconcentration. The platelet count nadir was defined as the lowest platelet value obtained.

#### Specific dengue tests

Acute (t0) and convalescent (t1 or t2) paired sera were tested by enzyme-linked immunosorbent assay (ELISA) using Dengue IgM-capture (PanBio, Ltd, Brisbane, Australia) and Dengue IgG Indirect (PanBio, Ltd, Brisbane, Australia) commercial kits. The NS1Ag test (Biorad Platelia™) and RT-PCR (Lanciotti et al., 1992) [19] were performed in acute serum samples. All of the tests were conducted according to the manufacturers’ instructions.

### Reverse Transcriptase – Polymerase Chain Reaction (RT-PCR)

Viral RNA was extracted from serum samples using a purification column from the PureLink™ Viral RNA/DNA Kit (Invitrogen, Carlsbad, CA). In one-step reverse transcription, cDNA was obtained and amplified using consensus primers to viral genome region (Capsid/prM). The nested PCR was performed to amplify the products of DNA using specific-serotypes primers to identify DENV-1 to 4 [19]. The amplicon were visualized on 1.2 % agarose gel electrophoresis according molecular size specific to each of the serotypes and stained with Gel Red (Biotium, BRA).

### Plaque reduction neutralization test (PRNT_50_)

Dengue serotype-specific neutralizing antibodies (NAbs) were determined in paired samples by a PRNT_50_ following a modified protocol by Morens et al*.* (1985) [20] and according to WHO guidelines [16]. In brief, the PRNT_50_ were conducted on Vero cells seeded at the density of 3 x 10^5^ cells/mL in MEM (Invitrogen, USA) with 10 % Fetal Bovine Serum (FBS) (GIBCO) in 24-well plates (0.5 mL/well) 24 h before assay. Serum samples were heat inactivated at 56 °C for 30 min than were diluted with MEM (1/20 to 1/2560) onto 96-well microtiter plates and incubation of 100 PFU of challenge virus. The assay was carried out using the DENV strains Brazil: DENV-1 (PE/97-42735), DENV-2 (PE/95-3808), DENV-3 (PE/02-95016) isolated in the State of Pernambuco, and DENV-4 isolated in the State of Roraima in 1982. After incubation for 1 h at 37 °C, 5 % CO_2,_ the medium was discharged and 50 μl of each dilution of the mixture serum/virus was inoculated in triplicate. The plates were then incubated at the same conditions to allow virus adsorption. The cells were covered with 500 μl of semi-solid medium and incubated for 7 days at 37 °C, 5 % CO_2_. After discarding the semi-solid medium, the cell monolayer was fixed with formalin, stained with crystal violet and plaques counted. We considered a sample as positive when NAbs levels were ≥1:20 against at least one serotype. The reciprocal of dilution of PRNT positivity was defined based on a 50 % reduction in plaque counts (PRNT_50_). To ensure accuracy and avoid inter-test variations, all of the procedures were performed by the same technician at a Public Health Laboratory Dr. Giovanni Cysneiros (LACEN-GO) in Goias state with technical supervision at the LaViTE, Centro de Pesquisas Aggeu Magalhães/FIOCRUZ in Recife, Pernambuco. The Brazilian institutions are part of the National Dengue Diagnosis Network.

### Definitions

The infecting serotype/current infection was defined as follows: a) ≥ 4-fold increase in serotype-specific NAbs titers among paired sera, or b) positive serotype-specific NAbs (PRNT_50_ ≥ 1/20) in a convalescent sample but as a negative result in acute sample. Moreover, the infecting serotype was also defined by RT-PCR in acute samples.

A monotypic response was defined by the presence of NAbs against only one of the four DENV serotypes. A multitypic response was defined as a concomitant detection of NAbs against two (dual), three or more serotypes.

A primary infection was defined by detecting NAbs against the infecting serotype in the absence of pre-existing NAbs in paired sera. A secondary infection was defined by detecting the infecting serotype and the presence of preexistent heterologous NAbs.

A sequential DENV infection was identified when there was seroconversion of NAbs for the infecting serotype and a detection of similar titers of heterologous NAbs in paired sera. It was not possible to determine the sequence of infections in the presence of NAbs against three or more serotypes.

### Statistical analyses

The main characteristics of the study population were described. The percentage of increase in hematocrit and the platelet count nadirs were stratified by severe and dengue cases. Albumin, AST and ALT values were categorized according to reference levels and compared among the dengue groups. The *X* ^*2*^ test was applied for categorical variables, t-test to detect difference between means, 2-tailed *p* < 0.05 was considered statistically significant.

The reciprocal of dilution of PRNT_50_ values were log-transformed (log base 10) after adding one (setting PRNT <1/20 to a value of 1). The geometric mean titers (GMT) and 95 % CIs were calculated. The median values of the PRNT titers (log base 10) difference among paired samples was compared using a non-parametric test of Friedman.

Statistical analyses was performed using SPSS v.18.0 (Inc., Chicago, IL, USA) and GraphPad Prism v.6.04 (GraphPad Software, USA)

## Results

Sixty subjects with paired samples of dengue confirmed cases were selected for the PRNT_50_ to all four DENV serotypes. The age range of severe dengue and dengue patients varied from 3 to 81 years old and from 11 to 79 years old, respectively, with a statistical significant difference in the mean ages between the groups (*t-test* = 5.26). There was a significant association between severity of dengue and hospitalization *X* ^*2*^ = 6.91; *p =* 0.02). Six patients had positive tourniquet test as the only hemorrhagic manifestation and were classified as dengue cases. Hemorrhagic manifestation was predominant among the severe dengue cases, a statistical significant association when compared to dengue cases (*X* ^*2*^ = 22.85; *p <* 0.01). When considering the platelet count nadir, the severe dengue cases had a higher frequency of low platelets levels (57.5 % ≤ 100 platelets/mm^3^) compared with 25 % in the dengue group, yielding a statistically significant association between the groups. An increase of 10 % or more in the hematocrit levels was not significantly different between the groups (*X* ^*2*^ = 1.60; *p =* 0.34). Higher frequencies of low albumin levels (<3.5 g/dL) were detected among the severe dengue cases (47.5 %) compared with the control group (20.0 %) (*X* ^*2*^ = 4.19; *p* = 0.04). Severe dengue cases presented higher values of AST compared with the control group (*X* ^*2*^ = 4.07; *p =* 0.04) (Table 1).

**Table 1:** Characteristics of dengue patients by disease severity during outbreak in central Brazil

Characteristics	Severe dengue^a^ *n* = 40	Dengue *n* = 20	*p* ^b^
Age, mean SD, years	33.6 ± 18.5	47.05 ± 20.5	<0.001
Sex, ratio of females to males	2.1:1.0	0.8:1.0	
Educational level, years^c^(%)			
≤ 8	17(51.5)	7(41.2)	0.49
> 8	16(48.5)	10(58.8)	
Race^d^, (%)			
White	18(48.7)	7(36.8)	0.46
Mixed/Black	19(51.3)	12(63.2)	
Hospitalization, (%)	20(50.0)	3(15.0)	<0.01
Hemorrhagic manifestation, (%)	36(90.0)	6^e^(30.0)	<0.01
Platelet count nadir, platelets/mm^3f^			
> 100	16(40.0)	15(75.0)	0.02
> 50 to ≤ 100	18(45.0)	5(25.0)	
≤ 50	6(15.0)	-	
Hematocrit increase, (%)			
< 10	32(80.0)	13(65.0)	0.35
≥ 10	8(20.0)	7(35.0)	
Albumin, (%)			
< 3.5 – low values	19(47.5)	4(20.0)	0.04
3.5–5.5 – normal values	21(52.5)	16(80.0)	
AST, (%)			
< 50 – normal values	13(32.5)	12(60.0)	0.04
≥ 50 – high values	27(67.5)	8(40.0)	
ALT, (%)			
< 41 – normal values	18(45.0)	11(55.0)	0.46
≥ 41 – high values	22(55.0)	9(45.0)	

Data are the no. (%) of subjects, unless otherwise indicated; *AST* aspartate aminotransferase, *ALT* alanine aminotransferase. Platelet count nadir was defined as the lowest platelet value obtained

^a^Clinically classified as dengue with warning signs or severe dengue

^b^ *X* ^*2*^ test was used for categorical variables

^c^Data were missing for seven severe dengue cases and three dengue cases

^d^Data were missing for three severe dengue cases and one dengue case

^e^Positive tourniquet test as the only hemorrhagic manifestation

^f^The platelet count nadir was defined as the lowest platelet value obtained

In total all patients presented NAbs for one or more DENV serotypes. Overall, 44 out of the 60 dengue patients (73.3 %) had NAbs to DENV-4, followed by DENV-1 (68.3 %), DENV-2 (68.3 %) and DENV-3 (61.6 %), considering each serotype per se. The majority of dengue cases independent of severity had multitypic infection (85 %). Nine out of the 40 severe dengue cases (22.5 %) had NAbs against all four DENV serotypes while seven out of 20 dengue cases (35.0 %) also had this multitypic response. There is no association between antibody response (monotypic, dual or multitypic) and severity of disease (X^*2*^ = 0.43; *p* = 0.81) (Table 2).

**Table 2:** Characteristics of serotype-specific neutralizing antibody response by PRNT_50_ of the dengue patients stratified by dengue severity

Characteristics	Severe dengue^a^ *n* = 40	Dengue *n* = 20
Antibody response		
Monotypic, (%)	6(15.0)	3(15.0)
DENV-1	2	1
DENV-2	-	2
DENV-3	-	-
DENV-4	4	-
Multitypic dual, (%)	11(27.5)	4(20.0)
DENV-1/DENV-2	4	-
DENV-1/DENV-3	-	1
DENV-1/DENV-4	1	-
DENV-2/DENV-4	1	3
DENV-3/DENV-4	5	-
Multitypic three or more, (%)	23(57.5)	13(65.0)
DENV-1/DENV-2/DENV-3	3	3
DENV-1/DENV-2/DENV-4	4	1
DENV-1/DENV-3/DENV-4	5	-
DENV-2/DENV-3/DENV-4	2	2
DENV-1/DENV-2/DENV-3/DENV-4	9	7

All of the patients had positive IgG results in acute or in convalescent serum samples according to inclusion criteria. 43 out of 60 subjects were IgM positive (71.6 %); 34 were NS1Ag positive (56.6 %). Twenty-four out of the 60 patients had a virological dengue infection confirmed by RT-PCR with the following results: DENV-4 (*n* = 14) and DENV-1 (*n* = 10) with negative virological results in 36 patients. At baseline, all of the patients had serum collected within 1–7 days, except one patient whose serum was collected at 9 days (median = 4.5 days). The range in the number of days for the serum collection in the follow-up period varied from 8 to 30 days with extreme values (7 days in 1 patient and 7 patients with a range of 31–44 days) (median = 21 days). The difference of the collection dates between convalescent and acute sera varied from 2 to 42 days (median = 16 days). Fifty-two out of 60 patients (86.6 %) had at least a four-fold increase in neutralizing antibodies in paired samples. Seroconversion by PRNT_50_ was not detected in the following eight patients: two DENV-1 infected cases (subjects 5 and 7) by RT-PCR and six (subjects 4, 30, 36, 39, 42, and 56) serologically-confirmed dengue cases (IgM and/or NS1Ag) presented with NAbs titers compatible with previous exposure without recognizing the infecting serotype. One patient who was 43 years old (subject 34) had DENV-1 and DENV-2 concurrent infections by NAbs seroconversion with type 1 confirmed by RT-PCR (Additional file 1: Table S1).

**Fig. 1:**
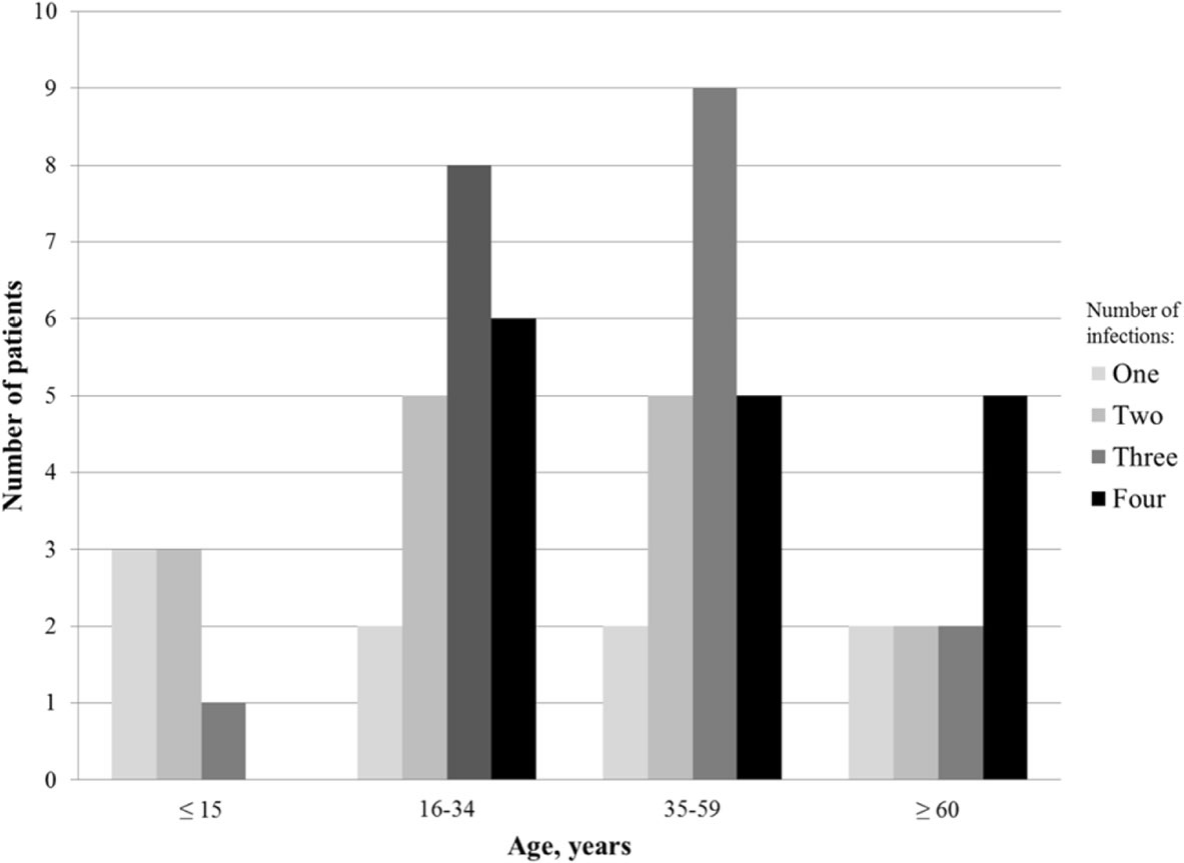
Neutralizing antibody response defined by PRNT_50_ to DENV-1 to 4 by age groups

Older age groups presented with higher frequencies of NAbs to three or more dengue serotypes compared with patients who were ≤ 15 years old. Serological evidence for exposure to all four dengue serotypes occurred in 16 out of 60 (26.6 %) participants. All of them were sixteen years old or more (Fig. 1). The youngest patient (3 years old) presented with a monotypic response with a seroconversion to serotype 4 by PRNT_50_ and had a confirmed DENV-4 infection by RT-PCR. A nine year old patient presenting with a DENV-4 infection by seroconversion had also pre-existing NAbs against serotype 3. Five dengue patients aged 10–15 years old had the following: monotypic response to DENV-4 (10 years old, subject 3); to DENV-2 (11 years old, subject 5); multitypic response to DENV-3/DENV-4 (11 years old, subject 4); DENV-2/DENV-4 (15 years old, subject 7) and response to DENV-2/DENV-3/DENV-4 (13 years old, subject 6) (Additional file 1: Table S1).

**Fig. 2:**
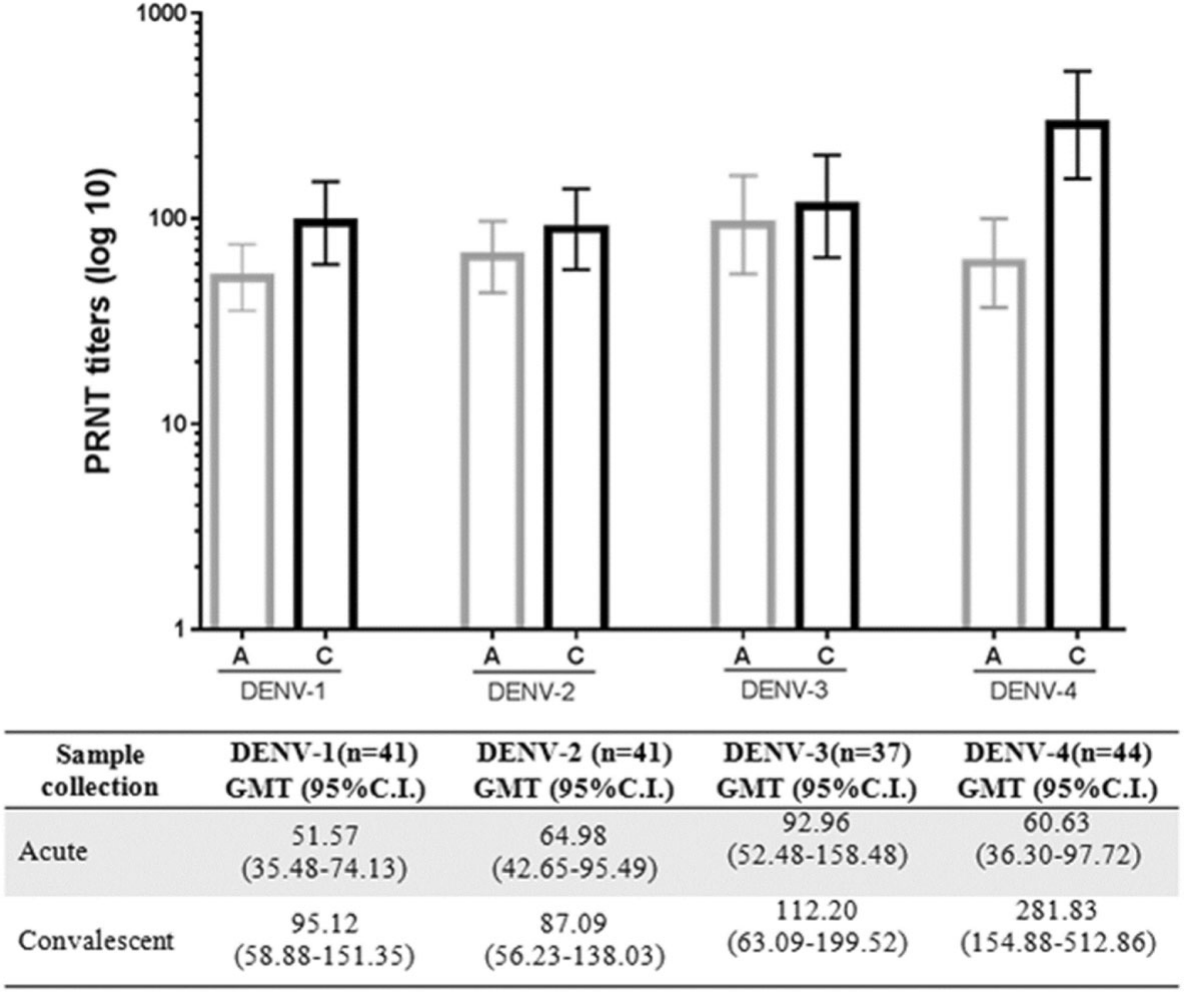
Representation of reciprocal PRNT_50_ titers in acute (A) and convalescent (C) sera. Neutralizing Abs titers to DENV-4 among paired samples (*t test p* < 0,001)

The geometric mean titers (GMT) values varied slightly from acute to convalescent sera with overlapping confidence intervals for DENV-1, 2 and 3. Neutralizing Abs titers to DENV-4 were higher in the convalescent sera (GMT = 281.83; IC95% 154.88-512.86) compared with the acute sera (GMT = 60.63; IC95% 36.30–97.72) (*p* < 0,001) (Fig. 2). We presented the box-plot distribution of the difference between the NAbs titers (log_10_) measured in convalescent and acute samples for all DENV serotypes (Friedman Test *X* ^*2*^ = 31.76; *p* < 0.001). The difference in the DENV-4 NAbs titers (log_10_) in paired samples varied from zero to 2.41; IQR (0.0–1.2) and median value of 0.60. Our results showed that the DENV-4 infected patients presented with the highest difference in PRNT_50_titers compared with the other serotypes (Additional file 2: Figure S1).

**Fig. 3:**
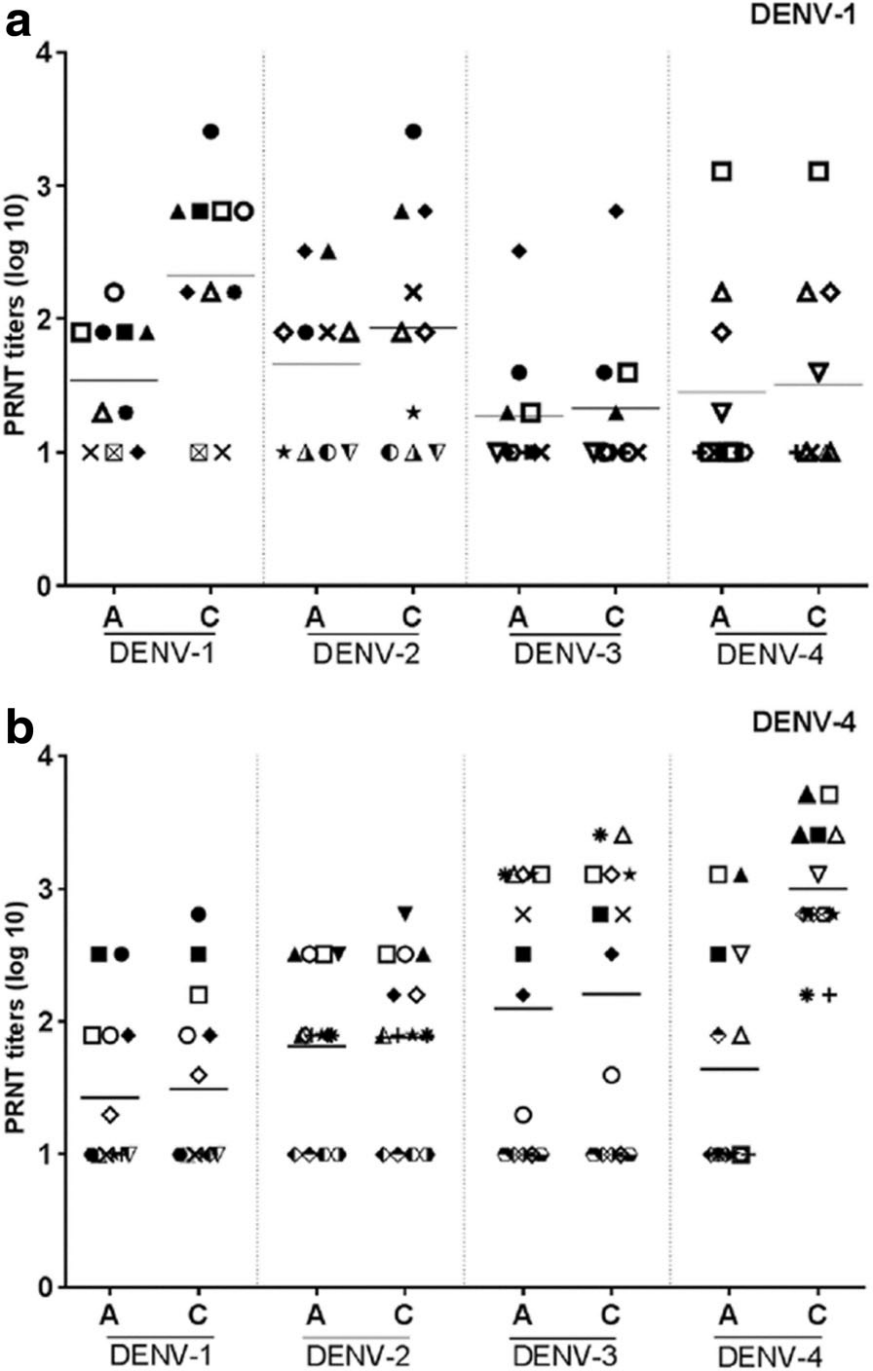
Graphical representation PRNT_50_ titers paired sera among patients infected by DENV-1 or DENV-4. Legend: PRNT_50_ titers (log10) in paired sera stratified for acute (A) or convalescent (C) phase of disease among patients infected by DENV-1 or DENV-4 determined by RT-PCR. (A) 10 patients infected by DENV-1; (B) 14 patients infected by DENV-4. Line represents mean

Figure 3 presents with individual NAbs titers stratified by DENV-1 or DENV-4 infected cases confirmed by RT-PCR. Seroconversion to serotype 4 by PRNT_50_ was detected among all DENV-4 virologically-confirmed patients. Among the 10 DENV-1 infected patients, eight also showed seroconversion to serotype 1_._ Two patients did not present with seroconversion in NAbs titers to serotype 1 probably due to the small lag time (≤7 days) between sample collections.

## Discussion

Our study showed evidence of the simultaneous circulation of all four DENV serotypes during the major outbreak in the year 2013 in central Brazil. The dengue patients presented with a high seroprevalence of neutralizing antibodies against DENV-1, DENV-2, DENV-3 and/or DENV-4 detected by PRNT_50_ mainly as a multitypic response. These results may reflect cumulative exposure to the circulating dengue virus in the study setting. In our study, DENV-4 was the predominant serotype during the outbreak in consonance with the main dengue virus isolated in the same period by surveillance data [8].

Most dengue patients presented with pre-existing NAbs levels for at least two DENV serotypes simultaneously. A multitypic response was predominant among children and adults suggesting early and continuous transmission of DENV serotypes in the study setting. Our results of a high frequency of multitypic response are in line with a previous population-based study in the northeast area of Brazil (2005–2006) [21].

In the present study, there was a high frequency of multitypic responses independently of the severity of the dengue cases. Tertiary or quaternary immune responses were similarly distributed between the severe dengue and control group.

One female adult (43 years old) recruited in the hospital was classified as a severe dengue case and had concurrent infections by DENV-1 and DENV-2 according to the PRNT_50_ results. DENV-1 was detected by RT-PCR, but DENV-2 was not, probably due to lower viral load of this serotype. The regional epidemiological scenario seems to be compatible with other studies that have also described the occurrence of concurrent dengue infections mainly during dengue outbreaks in hyperendemic areas with co-circulation of all four DENV serotypes in Asia [22–25] and in the Americas [26–28]. To our knowledge, this is the first concurrent dengue infection reported in Goias state, central Brazil.

Several recent studies have noted potential variations in PRNT_50_ assay such as virus strains, cell linage and other test components that may influence detection, quantification of NAbs titers with possible effects in PRNT_50_ results and comparability among studies [29–31]. In our study, we analyzed serotype-specific NAbs titers for the subset of patients with virological confirmation (RT-PCR). Subjects with the infecting DENV-1 or DENV-4 serotypes defined by PRNT_50_ had the same virological marker (DENV-1 or DENV-4) by RT-PCR suggesting the consistency of the assays performed. Our data also suggested a booster of pre-existing NAbs during a heterologous DENV infection as described in other studies [32, 33]. The need of paired samples to diagnose dengue infections by PRNT_50_ limits its application in clinical settings [1, 34, 35].

One of the constraints of the present study is the small sample size. PRNT consists of a laborious technique that requires a long time to run and technical skilled personnel, which limits its use in large-scale studies [16]. Our results represent a NAbs response in symptomatic dengue patients recruited in ambulatory and hospital settings, showing a point-of care situation. We are aware that our results should be interpreted with caution and may not be extrapolated to other Brazilian regions. However, this is a timely study that characterized the dengue specific-serotype immune response during a large dengue outbreak. It highlights the high frequency of multitypic response pattern in all age groups.

This paper adds knowledge about serotype-specific antibody profile in dengue patients during a DENV-4 outbreak in central Brazil. To our knowledge this is the first evidence of multiple dengue infections in a clinical cohort of severe and non-severe dengue cases in this setting.

## Conclusions

Our data indicates high exposure of multiple DENV serotypes in all age groups in the pre-dengue vaccine era and also previous to Zika virus introduction in Brazil. This is essential information for future dengue vaccines programs.

## Additional files

Additional file 1: Table S1. Individual data of patients according to specific dengue tests, serotype-specific reciprocal PRNT_50_ titers and clinical classification, during 2012-2013 outbreak in central Brazil, * Individual data of 60 patients according to PRNT_50_ titers and clinical classification, during 2012-2013 outbreak in central Brazil. (DOCX 56 kb)
Additional file 2: Figure S2. Difference between PRNT_50_ titers paired samples. Boxes encompass 50 % of the distribution; line represents median, * Difference between PRNT_50_ titers among paired samples related to DENV-1 to 4. (DOCX 74 kb)
