# Supplementary material for: High frequency of pre-existing neutralizing antibody responses in patients with dengue during an outbreak in Central Brazil
Source: BMC Infect Dis. 2016 Oct 7;16:546. doi: 10.1186/s12879-016-1867-6 (PMC5055662; doi:10.1186/s12879-016-1867-6)
Supplement: Additional file 1: Table S1. — Individual data of patients according to specific dengue tests, serotype-specific reciprocal PRNT50 titers and clinical classification, during 2012-2013 outbreak in central Brazil, * Individual data of 60 patients according to PRNT50 titers and clinical classification, during 2012-2013 outbreak in central Brazil. (DOCX 56 kb) [file 12879_2016_1867_MOESM1_ESM.docx]

**Supplementary table.** Individual data of patients according to specific dengue tests, serotype-

specific reciprocal PRNT_50_ titers and clinical classification, during 2012-2013 outbreak in central Brazil.

| No. | Age  (years) | Sample (days)^a^ | RT-PCR | NS1Ag | IgM | IgG | Reciprocal PRNT_50_ titers^b^ | | | | Clinical Type |
| --- | --- | --- | --- | --- | --- | --- | --- | --- | --- | --- | --- |
|  |  |  |  |  |  |  | DV-1 | DV-2 | DV-3 | DV-4 |  |
| 1 | 3 | S1 (5) | ND | - | + | + | 10 | 10 | 10 | **320** | Severe |
|  |  | S2 (17) |  |  |  |  | 10 | 10 | 10 | **2560** |  |
| 2 | 9 | S1 (6) | ND | - | + | + | 10 | 10 | 80 | **320** | Severe |
|  |  | S3 (19) |  |  |  |  | 10 | 10 | 160 | **2560** |  |
| 3 | 10 | S1 (3) | ND | + | - | - | 10 | 10 | 10 | **10** | Severe |
|  |  | S2 (37) |  |  |  | + | 10 | 10 | 10 | **160** |  |
| 4 | 11 | S1 (5) | ND | - | + | + | 10 | 10 | 320 | 80 | Severe |
|  |  | S2 (10) |  |  |  |  | 10 | 10 | 320 | 80 |  |
| 5 | 11 | S1 (2) | DV-1 | + | - | - | 10 | 10 | 10 | 10 | Dengue |
|  |  | S2 (9) |  |  |  | + | 10^c^ | 20 | 10 | 10 |  |
| 6 | 13 | S1 (7) | ND | - | + | + | 10 | 320 | 1280 | **1280** | Severe |
|  |  | S2 (13) |  |  |  |  | 10 | 320 | 1280 | **≥2560** |  |
| 7 | 15 | S1 (1) | DV-1 | + | I | + | 10 | 80 | 10 | 80 | Dengue |
|  |  | S2 (9) |  |  |  |  | 10^d^ | 160 | 10 | 160 |  |
| 8 | 16 | S1 (7) | ND | - | + | + | **320** | 320 | 1280 | 1280 | Severe |
|  |  | S2 (25) |  |  |  |  | **2560** | 320 | 1280 | 1280 |  |
| 9 | 16 | S1 (4) | ND | + | + | + | 10 | 80 | 320 | **80** | Severe |
|  |  | S2 (13) |  |  |  |  | 10 | 160 | 640 | **2560** |  |
| 10 | 16 | S1 (5) | DV-1 | + | + | + | **20** | 320 | 10 | 10 | Severe |
|  |  | S2 (39) |  |  |  |  | **160** | 640 | 10 | 10 |  |
| 11 | 19 | S1 (4) | ND | - | + | + | 80 | **20** | 10 | 320 | Severe |
|  |  | S2 (14) |  |  |  |  | 160 | **160** | 10 | 640 |  |
| 12 | 19 | S1 (6) | DV-4 | + | + | + | 320 | 320 | 160 | **80** | Dengue |
|  |  | S2 (10) |  |  |  |  | 320 | 640 | 320 | **640** |  |
| 13 | 20 | S1 (6) | ND | - | + | + | 320 | 160 | **320** | 1280 | Severe |
|  |  | S2 (18) |  |  |  |  | 320 | 160 | **2560** | 1280 |  |
| 14 | 20 | S1 (6) | ND | + | + | + | 1280 | 1280 | 20 | **80** | Severe |
|  |  | S2 (8) |  |  |  |  | 2560 | 2560 | 40 | **640** |  |
| 15 | 20 | S1 (2) | ND | + | - | - | 10 | 10 | 10 | **10** | Severe |
|  |  | S2 (44) |  |  |  | + | 10 | 10 | 10 | **40** |  |
| 16 | 21 | S1 (4) | ND | + | - | + | **80** | 640 | 80 | 10 | Severe |
|  |  | S2 (14) |  |  |  |  | **320** | 640 | 160 | 10 |  |
| 17 | 23 | S1 (6) | ND | + | + | + | 10 | 10 | 320 | **10** | Severe |
|  |  | S2 (24) |  |  |  |  | 10 | 10 | 320 | **160** |  |
| 18 | 23 | S1 (4) | DV-1 | - | I | + | **80** | 80 | 320 | 10 | Severe |
|  |  | S2 (19) |  |  |  |  | **640** | 80 | 640 | 10 |  |
| 19 | 23 | S1 (2) | DV-4 | - | - | - | 10 | 10 | 10 | **10** | Severe |
|  |  | S2 (21) |  |  |  | + | 10 | 10 | 10 | **160** |  |
| 20 | 23 | S1 (3) | ND | + | + | + | 20 | 10 | 1280 | **320** | Severe |
|  |  | S2 (10) |  |  |  |  | 40 | 10 | 1280 | **2560** |  |
| 21 | 23 | S1 (3) | DV-4 | - | + | + | 10 | 80 | 1280 | **320** | Dengue |
|  |  | S2 (28) |  |  |  |  | 10 | 160 | 1280 | **2560** |  |
| 22 | 26 | S1 (2) | DV-1 | + | - | + | **80** | 80 | 20 | 20 | Dengue |
|  |  | S2 (21) |  |  |  |  | **640** | 80 | 20 | 40 |  |
| 23 | 28 | S1 (5) | ND | + | + | + | **80** | 320 | 10 | 10 | Severe |
|  |  | S2 (28) |  |  |  |  | **640** | 640 | 10 | 10 |  |
| 24 | 28 | S1 (5) | DV-4 | + | + | + | 80 | 80 | 10 | **10** | Severe |
|  |  | S2 (31) |  |  |  |  | 80 | 80 | 10 | **640** |  |
| 25 | 32 | S1 (4) | ND | + | + | + | 80 | 10 | 10 | **80** | Severe |
|  |  | S2 (19) |  |  |  |  | 160 | 10 | 10 | **2560** |  |
| 26 | 32 | S1 (5) | ND | + | + | + | 320 | 10 | 320 | **20** | Severe |
|  |  | S2 (26) |  |  |  |  | 640 | 10 | 640 | **640** |  |
| 27 | 33 | S1 (5) | ND | + | + | + | **80** | 320 | 10 | 10 | Severe |
|  |  | S2 (21) |  |  |  |  | **320** | 640 | 10 | 10 |  |
| 28 | 33 | S1 (3) | DV-4 | + | + | + | 10 | 80 | 10 | **10** | Dengue |
|  |  | S2 (20) |  |  |  |  | 10 | 80 | 10 | **160** |  |
| 29 | 34 | S1 (4) | DV-4 | + | + | + | 80 | 320 | 1280 | **1280** | Severe |
|  |  | S2 (23) |  |  |  |  | 160 | 320 | 1280 | **≥2560** |  |
| 30 | 37 | S1 (4) | ND | + | I | + | 20 | 10 | 10 | 10 | Severe |
|  |  | S2 (7) |  |  |  |  | 20 | 10 | 10 | 10 |  |
| 31 | 39 | S1 (4) | ND | + | + | + | 80 | **80** | 1280 | 1280 | Dengue |
|  |  | S2 (31) |  |  |  |  | 80 | **640** | 1280 | 1280 |  |
| 32 | 41 | S1 (4) | ND | + | - | + | **40** | 80 | 320 | 10 | Dengue |
|  |  | S2 (23) |  |  |  |  | **160** | 160 | 640 | 10 |  |
| 33 | 42 | S1 (9) | ND | - | + | + | 80 | 10 | 1280 | **160** | Severe |
|  |  | S2(23) |  |  |  |  | 80 | 10 | 1280 | **2560** |  |
| 34 | 43 | S1 (4) | DV-1 | + | + | + | **80** | **80^e^** | 10 | 10 | Severe |
|  |  | S2 (12) |  |  |  |  | **2560** | **2560** | 10 | 10 |  |
| 35 | 43 | S1 (2) | DV-4 | + | - | - | 10 | 80 | 10 | **10** | Severe |
|  |  | S2 (29) |  |  |  | + | 10 | 80 | 10 | **640** |  |
| 36 | 44 | S1 (2) | ND | - | + | + | 160 | 80 | 20 | 10 | Dengue |
|  |  | S2 (21) |  |  |  |  | 160 | 80 | 40 | 10 |  |
| 37 | 45 | S1 (5) | DV-4 | - | + | + | 10 | 80 | 1280 | **320** | Dengue |
|  |  | S2 (30) |  |  |  |  | 10 | 160 | 2560 | **1280** |  |
| 38 | 47 | S1 (1) | ND | - | I | + | **20** | 20 | 1280 | 10 | Dengue |
|  |  | S2 (12) |  |  | + |  | **80** | 40 | 1280 | 10 |  |
| 39 | 47 | S1 (7) | ND | + | + | + | 640 | 640 | 1280 | 10 | Severe |
|  |  | S2 (9) |  |  |  |  | 640 | 640 | 1280 | 10 |  |
| 40 | 48 | S1 (3) | DV-1 | + | + | + | **160** | 10 | 40 | 160 | Severe |
|  |  | S2 (10) |  |  |  |  | **640** | 10 | 40 | 160 |  |
| 41 | 48 | S1 (6) | DV-4 | - | + | + | 320 | 320 | 1280 | **1280** | Severe |
|  |  | S2 (23) |  |  |  |  | 640 | 320 | 1280 | **≥2560** |  |
| 42 | 49 | S1 (5) | ND | - | I | + | 320 | 10 | 10 | 10 | Severe |
|  |  | S2 (21) |  |  | + |  | 640 | 10 | 10 | 10 |  |
| 43 | 50 | S1 (5) | ND | - | + | + | **80** | 320 | 1280 | 2560 | Severe |
|  |  | S2 (22) |  |  |  |  | **320** | 640 | 1280 | 2560 |  |
| 44 | 54 | S1 (7) | ND | - | + | + | 640 | 80 | 1280 | **20** | Severe |
|  |  | S2 (22) |  |  |  |  | 640 | 80 | 1280 | **640** |  |
| 45 | 55 | S1 (4) | DV-4 | + | + | + | 10 | 80 | 10 | **80** | Dengue |
|  |  | S2 (31) |  |  |  |  | 10 | 80 | 10 | **2560** |  |
| 46 | 56 | S1 (4) | ND | - | + | + | 80 | 320 | 10 | **80** | Severe |
|  |  | S2 (25) |  |  |  |  | 160 | 320 | 10 | **2560** |  |
| 47 | 56 | S1 (2) | DV-4 | + | - | + | 20 | 10 | 20 | **10** | Severe |
|  |  | S2 (23) |  |  |  |  | 40 | 10 | 40 | **2560** |  |
| 48 | 57 | S1 (6) | DV-1 | + | + | + | **20** | 10 | 20 | 10 | Dengue |
|  |  | S2 (19) |  |  |  |  | **160** | 10 | 40 | 10 |  |
| 49 | 57 | S1 (9) | ND | - | + | + | 320 | 640 | 1280 | **1280** | Dengue |
|  |  | S2 (37) |  |  |  |  | 320 | 640 | 1280 | **≥2560** |  |
| 50 | 60 | S1 (3) | DV-4 | + | - | + | 10 | 10 | 1280 | **10** | Severe |
|  |  | S2 (13) |  |  |  |  | 10 | 10 | 1280 | **640** |  |
| 51 | 62 | S1 (3) | DV-1 | + | - | - | **20** | 10 | 10 | 10 | Dengue |
|  |  | S2 (13) |  |  |  | + | **160** | 10 | 10 | 10 |  |
| 52 | 63 | S1 (5) | DV-1 | - | + | + | **80** | 320 | 10 | 1280 | Dengue |
|  |  | S2 (9) |  |  |  |  | **640** | 640 | 10 | 1280 |  |
| 53 | 65 | S1 (2) | DV-4 | + | I | + | 10 | 10 | 640 | **10** | Severe |
|  |  | S2 (27) |  |  |  |  | 10 | 10 | 640 | **640** |  |
| 54 | 66 | S1 (5) | ND | - | + | + | 320 | 320 | 10 | **640** | Severe |
|  |  | S2 (21) |  |  |  |  | 640 | 640 | 10 | **≥2560** |  |
| 55 | 71 | S1 (7) | ND | - | + | + | 320 | 320 | 160 | **160** | Severe |
|  |  | S2 (28) |  |  |  |  | 320 | 320 | 160 | **2560** |  |
| 56 | 71 | S1 (9) | ND | + | - | + | 10 | 80 | 10 | 10 | Dengue |
|  |  | S2 (23) |  |  |  |  | 10 | 80 | 10 | 10 |  |
| 57 | 76 | S1 (3) | DV-4 | + | I | + | 80 | 320 | 320 | **10** | Dengue |
|  |  | S2 (25) |  |  |  |  | 80 | 320 | 640 | **640** |  |
| 58 | 78 | S1 (5) | ND | - | + | + | 80 | 40 | 1280 | **320** | Dengue |
|  |  | S2 (28) |  |  |  |  | 80 | 40 | 1280 | **2560** |  |
| 59 | 79 | S1 (7) | ND | - | + | + | 320 | 320 | 1280 | **640** | Dengue |
|  |  | S2 (27) |  |  |  |  | 640 | 640 | 1280 | **≥2560** |  |
| 60 | 81 | S1 (5) | ND | - | + | + | 320 | 640 | 1280 | **640** | Severe |
|  |  | S2 (21) |  |  |  |  | 320 | 640 | 1280 | **2560** |  |

No: number of patient; S1: acute sample; S2: convalescent sample; DV-1: dengue virus 1; DV-2: dengue virus 2; DV-3: dengue virus 3; DV-4: dengue virus 4; RT-PCR: reverse transcriptase-polymerase chain reaction; NS1Ag: nonstructural 1 antigen; IgM: Immunoglobulin M; IgG: Immunoglobulin G; ND: non detectable. I: inconclusive; Severe includes dengue with warning signs and severe dengue cases; Dengue: dengue fever.

^a^Numbers in brackets indicate days of onset of symptoms. ^b^Numbers in bold indicate seroconversion determined by at least fourfold increase in titer of neutralizing antibody.
