# Supplementary figures and images for: High frequency of pre-existing neutralizing antibody responses in patients with dengue during an outbreak in Central Brazil
Source: BMC Infect Dis. 2016 Oct 7;16:546. doi: 10.1186/s12879-016-1867-6 (PMC5055662; doi:10.1186/s12879-016-1867-6)

**
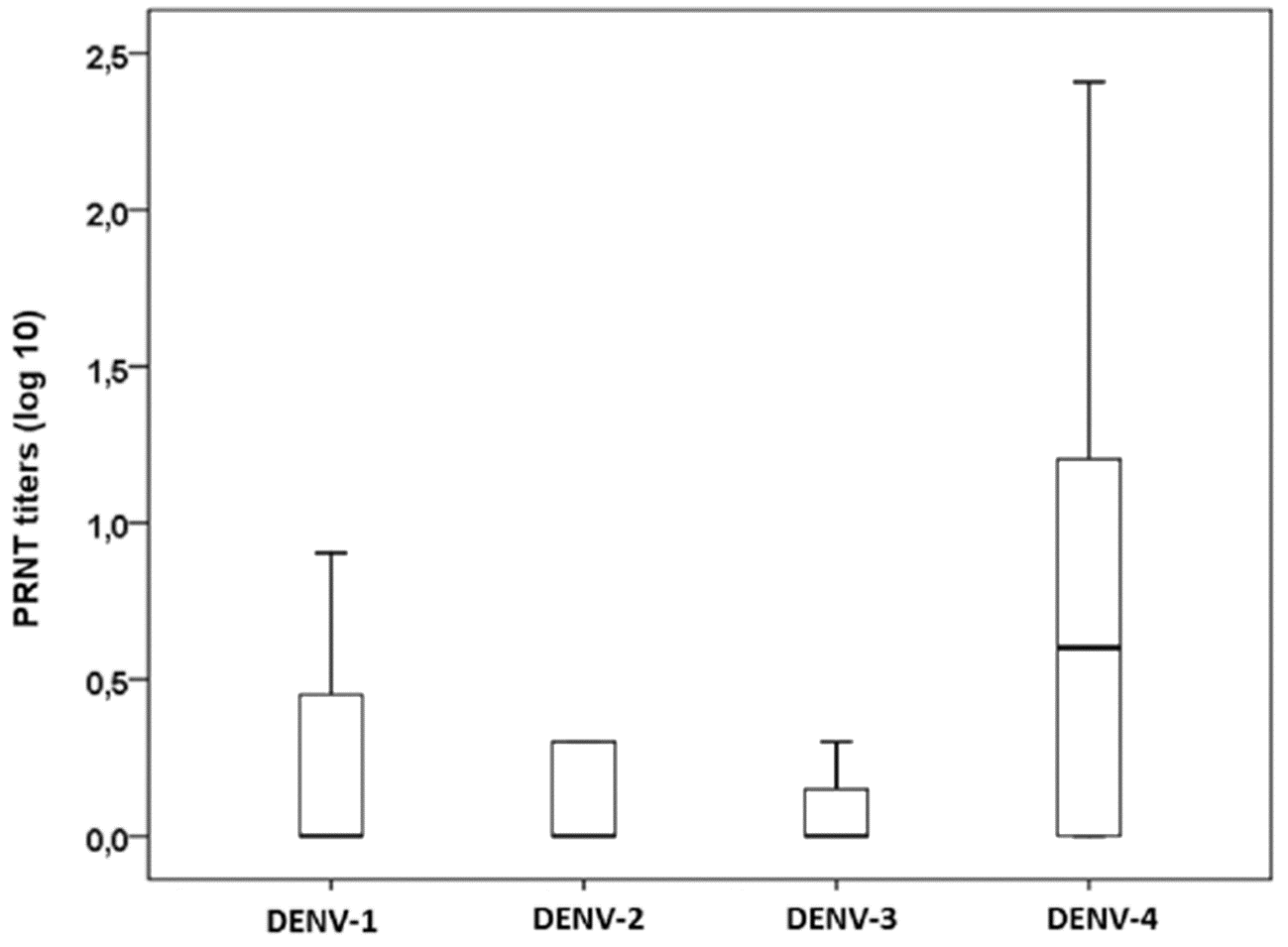
**

Supplement: Additional file 2: Figure S2. — Difference between PRNT50 titers paired samples. Boxes encompass 50 % of the distribution; line represents median, * Difference between PRNT50 titers among paired samples related to DENV-1 to 4. (DOCX 74 kb) [file 12879_2016_1867_MOESM2_ESM.docx]
